# Molecular Identification and Characterization of a Genotype 3 Hepatitis E Virus (HEV) Strain Detected in a Wolf Faecal Sample, Italy

**DOI:** 10.3390/ani11123465

**Published:** 2021-12-05

**Authors:** Vittorio Sarchese, Paola Fruci, Andrea Palombieri, Federica Di Profio, Serena Robetto, Carlo Ercolini, Riccardo Orusa, Fulvio Marsilio, Vito Martella, Barbara Di Martino

**Affiliations:** 1 Faculty of Veterinary Medicine, Università degli Studi di Teramo, 64100 Teramo, Italy; vsarchese@unite.it (V.S.); pfruci@unite.it (P.F.); apalombieri@unite.it (A.P.); fdiprofio@unite.it (F.D.P.); fmarsilio@unite.it (F.M.); 2 Istituto Zooprofilattico Sperimentale del Piemonte, Liguria e Valle d’Aosta, Centro di Referenza Nazionale per le Malattie degli Animali Selvatici (CeRMAS), 11020 Aosta, Italy; serena.robetto@izsto.it (S.R.); riccardo.orusa@izsto.it (R.O.); 3 Istituto Zooprofilattico Sperimentale del Piemonte, Liguria e Valle d’Aosta, SC Liguria e Portualità Marittima, 19100 La Spezia, Italy; carlo.ercolini@izsto.it; 4 Department of Veterinary Medicine, Università Aldo Moro di Bari, 70121 Valenzano, Italy; vito.martella@uniba.it

**Keywords:** *Orthohepevirus A*, HEV, genotype 3, wolf, stool sample

## Abstract

**Simple Summary:**

Data on hepatitis E virus (HEV) strains circulating in carnivores are still limited. However, serological evidence suggests that their exposure to HEV infection is more common than expected. In addition, close relatives of *Orthohepevirus C* detected in rats and in human patients with persistent hepatitis have been detected in ferrets, minks, and foxes, providing a rationale for investigating the presence of HEV or HEV-like viruses in wild carnivores. Herewith, we report the identification and the nearly full-genome (7212 nucleotides) characterization of an HEV Gt3 strain detected in a faecal specimen collected from a wolf found dead in Northwestern Italy. This finding poses questions concerning the potential role of wolves as HEV mechanical, passive carriers, favouring the introduction and spread of HEV in the environment.

**Abstract:**

Hepatitis E virus (HEV) infection is a major health problem worldwide. In developed countries, zoonotic transmission of HEV genotypes (Gt) 3 and 4 is caused by the ingestion of raw or undercooked meat of infected pigs and wild boars, the main reservoirs of HEV. However, additional animals may harbour HEV or HEV-related strains, including carnivores. In this study, we investigated the molecular epidemiology of orthohepeviruses in wild canids by screening a total of 136 archival faecal samples, collected from wolves (42) and red foxes (94) in Northwestern Italy. Orthohepevirus RNA was identified in a faecal specimen, collected from a wolf carcass in the province of La Spezia (Liguria Region, Italy). The nearly full-length (7212 nucleotides) genome of the strain HEV/81236/Wolf/2019/ITA (GenBank accession no. MZ463196) was determined by combining a sequence-independent single-primer amplification (SISPA) approach with the Oxford Nanopore Technologies sequencing platform. Upon phylogenetic analysis, the HEV detected in wolf was segregated into clade HEV-3.1, displaying the highest nucleotide (nt) identity (89.0–93.3%) to Gt3 strains belonging to subtype c. Interestingly, the wolf faecal sample also contained porcine astrovirus sequences, endorsing the hypothesis of a dietary origin of the HEV strain due to preying habits.

## 1. Introduction

Hepatitis E virus (HEV) is a common cause of human acute viral hepatitis [1]. HEV is a single-stranded, positive-sense RNA virus of about 6.4–7.2 kb in length, organized in three open reading frames (ORFs) [2] and classified in the family *Hepeviridae* [3]. In the last few years, identification of genetically divergent HEV strains has suggested an update of the classification of the family *Hepeviridae* into two genera, namely the genus *Piscihepevirus* (cutthroat trout virus) and *Orthohepevirus* [3]. The genus *Orthohepevirus* is further subdivided into four distinct species, *Orthohepevirus A–D,* including hepeviruses of mammalian and avian origin [4]. Based on the full-length genome analysis, HEV strains within the species *Orthohepevirus A* have been assigned to eight distinct genotypes (Gt1-Gt8) [5], with four major Gts (1–4) implicated in human infection/disease. Gt1 and Gt2 are endemic in South and Southeast Asia, Africa, and Mexico and restricted to humans, where they are predominantly transmitted through the faecal–oral route, either indirectly through contaminated drinking water or food [1]. Gt3 and Gt4 are considered zoonotic, and they are responsible for sporadic cases and outbreaks of hepatitis E in industrialized countries, chiefly due to ingestion of raw or undercooked meat products. Pigs and wild boars represent the primary reservoirs of infection [6], although recent identification of Gt3 and Gt4 strains from various animal species has significantly broadened the host range of *Orthohepevirus A* [7]. HEV infection has also been documented in wild carnivores [8,9,10,11,12,13]. Initial evidence was collected in 2002 by testing sera from mongoose captured in Okinawa prefecture (Japan) [8]. HEV IgG antibodies were found in 21.0% of the tested animals, and HEV RNA was detected in one mongoose (1.0%). Sequencing of the full genome characterised the virus as Gt3 [8]. Subsequently, similar HEV strains have been identified in 2.9% of mongoose bile samples from the same region [10]. Additionally, Gt4 HEV strains have been detected in two faecal samples collected respectively from an Asiatic black bear and a clouded leopard housed in a wildlife first-aid centre in Eastern China [9]. In addition to the species *Orthohepevirus A*, hepeviruses closely related to rat orthohepeviruses C (HEV-C1) [14], but clustering into a distinct genotype (HEV-C2), were detected in 2012 by metagenomic investigation in faecal samples from household pet ferrets in the Netherlands [11]. Since then, carnivore-derived HEV-C2 strains have been detected in liver and faecal specimens from farmed American minks in Denmark [13] and in faeces of red foxes in the Netherlands, Germany, and Hungary [12,15,16].

In this study, faecal samples from wolves (*Canis lupus italicus*) and red foxes (*Vulpes vulpes*) were analysed molecularly, using primer sets targeting highly conserved regions among the four major Gts (Gt1-Gt4) of *Orthohepevirus A* and a primer set designed to amplify all members of the genus *Orthohepevirus*. The nearly full genome sequence of an HEV Gt3 strain detected in a wolf faecal sample was characterized.

## 2. Materials and Methods

### 2.1. Sampling

Molecular investigation for orthohepeviruses was performed on archived rectal swabs collected from 42 wolves found dead in Piemonte (33), Liguria (6), and Valle D’Aosta (3) between February 2017 and September 2020. Additionally, in the analysis were included 94 red foxes found dead or shot during the regular hunting season in the time frame September 2013–November 2017.

### 2.2. Molecular Screening for Orthohepeviruses

Total RNA was extracted from each faecal specimen (0.5 mL) by using the TRIzol LS (Invitrogen, Ltd., Paisley, UK) procedure and analysed by *Orthohepevirus A*-specific real-time reverse transcription PCR (qRT-PCR) [17]. Tenfold serial dilutions (from 10^7^ to 10^2^ copies) of a plasmid containing the 68-nt ORF3 fragment of a Gt3 HEV wild boar strain (HEV/WB/P6-15/ITA, accession no. KU508285) [18] were used in each PCR run. The first WHO international standard for HEV RNA (code 6329/10) was used to standardize the system. Viral RNA quantification was performed using the SuperScript III platinum OneStep Quantitative RT-PCR system (Invitrogen Ltd., Milan, Italy) in a 25 μL volume comprising 5 μL of extracted RNA and 20 μL of master mix. Primers and TaqMan probe were used at concentrations of 200 and 100 nM, respectively. Screening for other members of the genus *Orthohepevirus* was done by a pan-orthohepevirus heminested RT-PCR using broadly reactive primers designed to amplify all members of the genus *Orthohepevirus* [19] and targeting a region of 338 nt of the viral RNA-dependent RNA polymerase (RdRp) coding sequence. The sample HEV-RNA positive with the above molecular strategies was further tested by a nested RT-PCR targeting the ORF2 gene [20]. The positive amplicons were excised from the gel, purified using the QIAquick gel extraction kit (Qiagen GmbH, Hilden, Germany), and then subjected to direct sequencing using BigDye Terminator Cycle chemistry and 3730 DNA Analyzer (Applied Biosystems, Foster, CA, USA). Basic Local Alignment Search Tool (BLAST; http://www.ncbi.nlm.nih.gov, accessed on 10 October 2021) and FASTA (http://www.ebi.ac.uk/fasta33, accessed on 10 October 2021) with default values were used to find homologous nucleotide sequences. 

### 2.3. Whole Genome Sequencing

A sequence-independent single-primer amplification (SISPA) approach [21] combined with Oxford Nanopore Technologies (ONT) sequencing was used to generate HEV full-length genome and to further investigate the virome composition of the wolf gut content. Briefly, DNase-treated RNA was reverse transcribed to single strand cDNA by SuperScript IV Reverse Transcriptase (Invitrogen Ltd, Milan, Italy) using primers FR26RV-N (GCCGGAGCTCTGCAGATATC-N6) [22] and FR40RV-T (GCCGGAGCTCTGCAGATATCTTTTTTTTTTTTTTTTTTTT) [23]. Second strand cDNA synthesis was performed using Klenow Fragment (3'→5' exo-) (New England BioLabs, Hitchin, UK). Primer FR20RV (GCCGGAGCTCTGCAGATATC) [22] was used to generate the cDNA. The PCR product was cleaned up with AMPure XP beads (Beckman Coulter, USA) and quantified with a Qubit 4.0 instrument using a dsDNA HS Assay Kit (Thermo Fisher Scientific, Waltham, MA, USA). Nanopore sequencing library preparation was performed according to manufacturer’s instructions for Ligation Sequencing Kit (SQK-LSK110, Oxford Nanopore Technologies). Briefly, DNA ends were end-repaired and dA-tailed using the NEBNext End Repair/dA tailing module (New England Biolabs, Hitchin, UK) and ligated to sequencing adaptor. Nanopore library was run for nine hours on Oxford Nanopore MinION Mk1C device using FLO-MIN106 R9.4.1 flow cell after loading 75 μL sequencing mix (12 μL library, 25.5 μL Loading Beads II, 37.5 μL Sequencing Buffer II). Primary data acquisition into FAST5 files was carried out using MinKNOW software (Oxford Nanopore Technologies, Oxford, UK). Raw sequence reads were uploaded to the EPI2MEAgent Software v3.3.0 interface (Metrichor, Oxford, UK), which was used for base-calling and quality score. Base-called data passing parameters were downloaded in FASTQ format. Sequence trimming, assembly of reads, and genome annotation were performed using the Geneious Prime version 2021.1.1 (Biomatters Ltd., Auckland, New Zealand). The reads classified in the EPI2MEAgent report were mapped and assembled by using Minimap2 v2.17 plugin of the Geneious software. The resulting draft consensus sequences were subjected to BLASTn search. The alignment of the sequences was conducted using the MAFFT multiple alignment program version 7.388 plugin of the Geneious software. Phylogenetic analysis was conducted using Maximum Likelihood, Tamura-Nei model, supplying statistical support with bootstrapping of 1000 replicates, in MEGA X software [24].

## 3. Results

**Figure 1:**
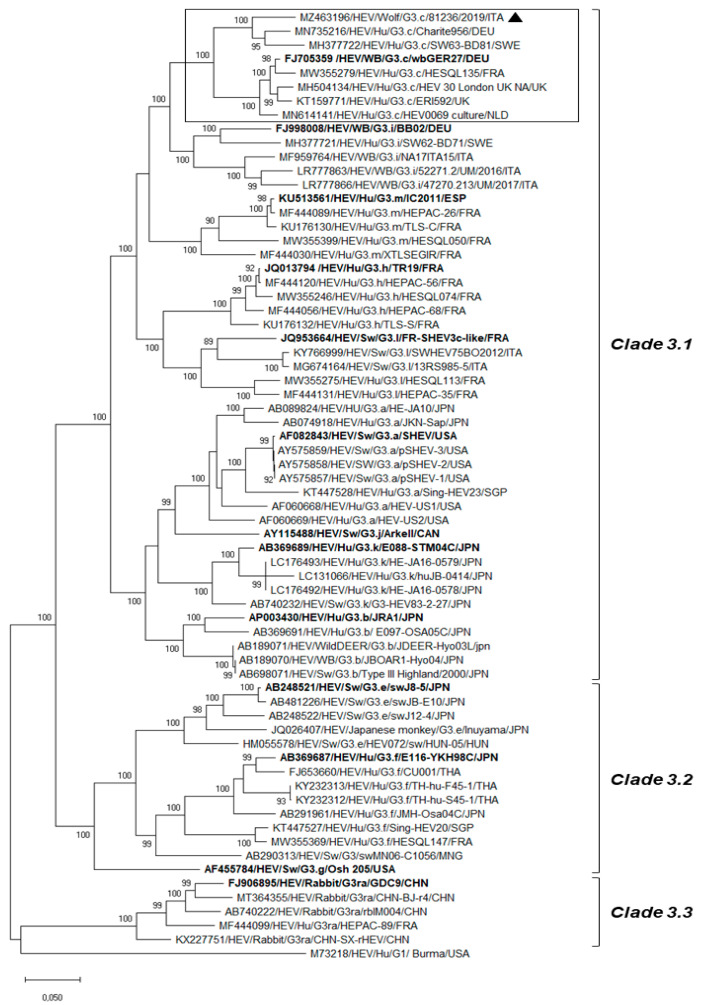
Phylogenetic analysis performed on the complete nucleotide sequence (7212 nt) of the strain HEV/81236/Wolf/2019/ITA (GenBank accession no. MZ463196). Tree was generated using maximum likelihood method based on the Tamura–Nei model and supplying statistical support with bootstrapping of 1000 replicates. A selection of complete genome sequences of the *Orthohepevirus A* species, including all the reference strains [5] representative of each HEV Gt3 subtype group (http://talk.ictvonline.org accessed on 10 October 2021), was used for the analysis. The scale bar indicates nucleotide substitutions per site. The reference strains representative of each HEV Gt3 subtype are in boldface. HEVs classified within the subtype c were delimited by rectangle. The marker denotes the HEV sequence detected in this study. Evolutionary analyses were conducted in MEGA X [24].

Out of 136 faecal samples collected from wild canids, qRT-PCR detected HEV RNA with a viral load of 2.1 × 10^3^ RNA copies/gram faeces (Ct 28.00; cut-off Ct 40.00) in one specimen (0.73%; 1/136) collected from an adult male wolf (2.3%; 1/42) found dead in La Spezia province, (Liguria Region). The cause of death was trauma with organ rupture and exsanguination likely due to car accident. The same specimen resulted positive when re-screened with a pan-orthohepevirus broadly reactive RT-PCR [19] and with primer specific for a 348-nt ORF2 region of Orthohepevirus A [20]. BLAST and FASTA (accessed on 10 October 2021) analyses of the partial RdRp and ORF2 regions revealed the highest nucleotide identity to HEV strains classified into Gt3 within the species Orthohepevirus A (nt identities of 80.3–97.5% and 80.2–94.0%, respectively). Nucleotide identities to hepeviruses of species Orthohepevirus B, C, D ranged from 58.8% to 63.8% in the RdRp region and from 52.9% to 63.2% in the ORF2 gene. The ONT sequencing platform was successfully used to generate the full-length genome of the wolf strain HEV/81236/Wolf/2019/ITA (GenBank accession no. MZ463196). Out of a total output of 480,834 raw nanopore reads, 210,000 were base-called with an average length of 900 nt (range 100–4400 nt). Sequencing data were filtered to remove low-quality reads using a quality score threshold of seven. Furthermore, reads shorter than 300 nt were removed and not included in the analysis. In total, 3499 reads were classified as orthohepevirus sequences. Additional detected viral sequences aside from HEV corresponded to lambda phage control spike-in (36,000 reads) and mamastroviruses (288 reads), of which most (177 reads) showed the highest identity (90.0% nt) to a wild boar astrovirus strain (WBAstW-1/2011/HUN; GenBank accession no. JQ340310) [25]. The strain HEV/SwGt3/Meng (GenBank accession no. AF082843) was used to perform a reference-based assembly by Minimap2. The long reads (mean read length 1910 nt and mean quality of 9.6) covered almost the entire genome (99.88%) of the Gt3 Meng strain (only nine nucleotides missing at the 5’UTR) with an average coverage depth of 65x, producing a unique consensus sequence of 7212 nt. Three ORFs were predicted from computer analysis of the nucleotide sequence and by comparing the results with the genomic organization and ORFs of other Gt3 HEVs. ORF1 was 5112 nt long (nt 1–5112) and has the capacity to encode a polyprotein of 1703 amino acids (aa), ORF2 was 1983 nt in length (nt 5147–7129) and has the capacity to encode a capsid protein of 660 aa, and ORF3 was 369 nt long (nt 5109–5477) and has the capacity to encode a protein of 122 aa. Upon sequence analysis of the nearly complete genome, the highest sequence matches (92.3–93.3% nt identity) were found to the strain Charite956/DEU/2009 (GenBank accession no. MN735216) (E-value: 0.0; max and total scores 10648) detected in the liver sample of a Dutch patient with biochemical graft dysfunction (unpublished data) and to the HEV SW63-BD81 (GenBank accession no. MH377722) (E-value: 0.0; max and total scores 10272) identified in a plasma donor in Sweden [26]. Upon phylogenetic analysis (Figure 1), the HEV 81236/Wolf/2019/ITA was segregated into the clade HEV-3.1 [5,27,28], displaying the highest identity (89.0–93.3% nt) to strains classified within the Gt3 subtype c. Nucleotide identity to the prototype strain wbGER27 (GenBank accession no. FJ705359) was 89.1%, while identities to other subtypes ranged from 76.0% (Gt3ra) to 85.7% (Gt3i) (Table 1).

## 4. Discussion

Since the initial discovery of animal HEV and the understanding of its zoonotic potential [29], the knowledge of HEV epidemiology has been considerably increased. Furthermore, taking advantage of massive sequencing technologies for virus characterization and discovery, a number of novel HEV strains have been identified from a large diversity of mammalian species, largely expanding the host range of the species *Orthohepevirus A* [7]. Besides wild boars, recognized as the wildlife reservoir of zoonotic HEV [6], natural orthohepevirus infections have been occasionally reported in a variety of wild animals, including carnivores such as mongooses, bears, leopard, ferrets, minks, and foxes [8,9,10,11,12,13]. We undertook this study to better understand the ecology of orthohepeviruses in Italian wolves and red foxes implementing the HEV diagnostic algorithm with a pan-orthohepeviruses molecular strategy [19] that has also been previously used to detect HEV-C2 in red foxes [16]. In our analysis, orthohepevirus RNA was identified in a wolf faecal sample (81236/Wolf/2019/ITA), and, unexpectedly, we observed that it displayed the highest genetic relatedness to HEV strains classified as Gt3 within the species *Orthohepevirus A*. The SISPA technique in combination with ONT, a third-generation sequencer, which recently emerged as complement to or replacement for second-generation sequencers, was applied to target viral RNA sequences contained in the wolf faecal sample. This method allowed for successful sequence assembly of the nearly full genome of the strain HEV/81236/Wolf/2019/ITA. Upon visual inspection of the complete genome based-tree, consistent with previous analyses [5,27,28], three major clades were identified: clade-3.1 that includes subtypes a, b, c, h, k, i, j, l, and m; clade-3.2 which contains subtypes e, f, and g; and clade-3.3 that comprises HEV strains identified in rabbits (Gt3ra subtype). Strictly following the novel proposed classification criteria for HEV Gt3 [5,28], the HEV strain identified in this study was assigned to subtype c. Phylogenetic and coalescence analyses based on the partial ORF2 gene of HEV Gt3 strains identified in domestic pigs and wild boars in Italy in the time frame 2002–2017 provided evidence that subtypes 3c and 3f are the most common [30]. In addition to the number of studies reporting the circulation of HEV Gt3c in wild boars and pigs in Northwestern Italy [31,32,33], a Gt3 strain (PeGe, GenBank accession no. KF751185) genetically related to subtype c has been previously identified in a patient with acute hepatitis E in the Liguria Region (Genoa city), with suspected foodborne transmission [34]. Accordingly, the identification of the subtype c in the wolf faecal specimen seems to mirror the HEV subtype circulation observed in humans and animals in the same geographical area. 

To date, there is no clear evidence that zoonotic HEVs may infect or cause disease in canids. Experimental inoculation of two dogs with a swine Gt4 strain was able to elicit specific IgG antibodies, but HEV RNA was not found in infected animals [35]. Overall, the frequent detection of HEV-specific antibodies either in domestic or wild canids seems to suggest that abortive or subclinical infections might occur [36,37,38]. Herein, although we obtained a direct piece of evidence concerning the presence of an HEV Gt3 strain in the wolf intestinal content, the unavailability of additional samples to analyse, such as serum and liver, make it difficult to speculate on the true *Orthohepevirus A* susceptibility of this wild canid. Meanwhile, we cannot exclude its potential role as an HEV mechanical, passive carrier, likely due to preying on actively infected hosts. The wolf HEV-positive stool was collected in a geographical area (La Spezia, Liguria Region) characterized by a high density of wild boar populations in which HEV Gt3 subtypes 3a, 3c, 3e, and 3f have been found to circulate with prevalence rates ranging from 2.3% to 4.4% [32,33]. In our analysis, the employment of a random amplification protocol for viral RNA genomes prior to the application of ONT sequencing revealed that the wolf faecal sample also contained the reads genetically closest to wild boar astrovirus sequences. Accordingly, we made attempts to investigate the presence of the porcine DNA in the wolf intestinal content with primers specific for *Sus scrofa* [39], but it was not identified. Meanwhile, we cannot exclude that the presence of HEV Gt3 in the wolf intestinal content was due to preying on other actively infected animals such as pigs and wild and domestic ruminants [6,40,41,42].

## 5. Conclusions

In conclusion, the findings of this study pose questions concerning the role of wolves as potential HEV carriers, thus sustaining viral circulation in the ecosystem. Large-scale serological and molecular investigations in wolves and red foxes could be useful for carefully assessing their role in the HEV epidemiological cycle, chiefly in geographical settings in which animal reservoirs and predators live in sympatry. Furthermore, upon nearly complete genome characterization by using a third-generation sequencer, it was clear that HEV Gt3 strains of subtype c are circulating in the surveyed geographical area (Northwestern Italy), confirming previous studies [31,32,33]. A limitation of this study was that many of the human and non-human animal Italian HEV sequences, belonging to subtype c, are partial short fragments (~300 nt) of the ORF2 gene. Accordingly, it was not possible to look for possible genetic hallmarks in the HEV genome obtained from wolf faecal samples. Generating a large dataset of HEV Gt3 complete genome sequences will be pivotal for a more precise comparison of strains circulating in humans and non-human animals and for epidemiological tracing of the infection source.

## Figures and Tables

**Table 1:** Sequence identity of the HEV/81236/Wolf/2019/ITA (GenBank accession no. MZ463196) to prototype HEV strains representative of each Gt3 subtype for which the complete genome is currently available.

Subtype	Strain Name	ORF1	ORF2	ORF3	GenBank Accession no.
3a	Meng	83.6%	85.6%	92.7%	AF082843
3b	JRA1	83.5%	85.9%	94.0%	AP003430
3c	wbGER27	88.8%	90.6%	94.0%	FJ705359
3e	swJ8-5	79.4%	84.1%	93.5%	AB248521
3f	E116-YKH98C	79.7%	83.8%	94.0%	AB369687
3g	Osh 205	80.3%	84.1%	93.2%	AF455784
3h	TR19	84.6%	86.7%	94.0%	JQ013794
3i	BB02	85.0%	87.8%	95.7%	FJ998008
3j	Arkell	82.4%	86.0%	93.0%	AY115488
3k	E088-STM04C	83.6%	86.4%	95.1%	AB369689
3l	FR-SHEV3c-like	84.1%	86.2%	93.2%	JQ953664
3m	IC2011	84.0%	87.3%	93.5%	KU513561
3ra	GDC9	74.9%	81.6%	88.1%	FJ906895

## Data Availability

The sequence that supports the finding of this study is openly available in the GenBank database at https://www.ncbi.nlm.nih.gov/nucleotide/ under accession number MZ463196.
